# Splicing Dysregulation of Non-Canonical GC-5′ Splice Sites of Breast Cancer Susceptibility Genes *ATM* and *PALB2*

**DOI:** 10.3390/cancers16213562

**Published:** 2024-10-22

**Authors:** Inés Llinares-Burguet, Lara Sanoguera-Miralles, Alberto Valenzuela-Palomo, Alicia García-Álvarez, Elena Bueno-Martínez, Eladio A. Velasco-Sampedro

**Affiliations:** Splicing and Genetic Susceptibility to Cancer, Unidad de Excelencia Instituto de Biomedicina y Genética Molecular (IBGM) de Valladolid, Consejo Superior de Investigaciones Científicas-Universidad de Valladolid (CSIC-UVa), 47003 Valladolid, Spain; ines.llinares@estudiantes.uva.es (I.L.-B.); lara.sanoguera@uva.es (L.S.-M.); valenzuela.larioja@gmail.com (A.V.-P.); aliciaga@uva.es (A.G.-Á.); elena.bueno@uva.es (E.B.-M.)

**Keywords:** hereditary breast/ovarian cancer, *ATM*, *PALB2*, *BRIP1*, VUS, splicing, non-canonical splice sites, aberrant splicing, minigenes, variant classification

## Abstract

**Background/Objectives**: The non-canonical GC-5′ splice sites (5′ss) are the most common exception (~1%) to the classical GT/AG splicing rule. They constitute weak 5′ss and can be regulated by splicing factors, so they are especially sensitive to genetic variations inducing the misrecognition of their respective exons. We aimed to investigate the GC-5′ss of the breast/ovarian cancer susceptibility genes, *ATM* (exon 50), *BRIP1* (exon 1), and *PALB2* (exon 12), and their dysregulation induced by DNA variants. **Methods**: Splicing assays of the minigenes, mgATM_49-52, mgBRIP1_1-2, and mgPALB2_5-12, were conducted to study the regulation of the indicated GC-5′ss. **Results**: A functional map of the splicing regulatory elements (SRE) formed by overlapping exonic microdeletions revealed three essential intervals, *ATM* c.7335_7344del, *PALB2* c.3229_3258del, and c.3293_3322del, which are likely targets for spliceogenic SRE-variants. We then selected 14 *ATM* and 9 *PALB2* variants (Hexplorer score < −40) located at these intervals that were assayed in MCF-7 cells. Nine *ATM* and three *PALB2* variants affected splicing, impairing the recognition of exons 50 and 12, respectively. Therefore, these variants likely disrupt the active SREs involved in the inclusion of both exons in the mature mRNA. DeepCLIP predictions suggested the participation of several splicing factors in exon recognition, including SRSF1, SRSF2, and SRSF7, involved in the recognition of other GC sites. The *ATM* spliceogenic variants c.7336G>T (p.(Glu2446Ter)) and c.7340T>A (p.(Leu2447Ter)) produced significant amounts of full-length transcripts (55–59%), which include premature termination stop codons, so they would inactivate ATM through both splicing disruption and protein truncation mechanisms. **Conclusions**: *ATM* exon 50 and *PALB2* exon 12 require specific sequences for efficient recognition by the splicing machinery. The mapping of SRE-rich intervals in minigenes is a valuable approach for the identification of spliceogenic variants that outperforms any prediction software. Indeed, 12 spliceogenic SRE-variants were identified in the critical intervals.

## Simple Summary

*ATM* and *PALB2* are two of the main breast cancer susceptibility genes. In this research, we studied a crucial biological process called splicing that is responsible for removing introns and consecutively assembling exons, which contain the protein-coding sequence. We focused on mapping the splicing regulatory elements involved in the recognition of *ATM* exon 50, *BRIP1* exon 1, and *PALB2* exon 12, which present atypical GC-5′ splice sites. For this purpose, we used the biotechnological tool of hybrid minigenes, which mimic the splicing process of the gene of interest. Thus, we discovered one regulatory interval in *ATM* exon 50 and two in *PALB2* exon 12 that constitute hotspots where splicing-disrupting variants can be placed. Then, 23 *ATM* and *PALB2* candidate variants were tested. Nine *ATM* and three *PALB2* variants impaired splicing, affecting the recognition of their corresponding exons. These variants may therefore be associated with increased breast cancer risk.

## 1. Introduction

Splicing is a highly regulated gene expression step that is mediated by a wide array of splicing factors and sequence motifs that include the 5′ and 3′ splice sites (ss or donor and acceptor sites, respectively) and splicing regulatory elements (SRE), namely the exonic and intronic splicing enhancers (ESE/ISE) or silencers (ESS/ISS). These elements are involved in controlling splicing events, such as the recognition of alternative exons, non-canonical splice sites and small and large exons, among others [1,2,3,4]. Thus, variants at these elements may dysregulate the splicing patterns and be associated with a particular disease.

GC-5′ss, where the + 2T is replaced by C, is the most common exception to the canonical GT–AG rule, accounting for about 0.9% of all human exons [5]. GC-donors appear in alternative and constitutive exons where they show strong consensus sequences surrounding the GC dinucleotide [2,5]. On the other hand, alternative GC-exons show lower sequence conservation, and their identification would also require the intervention of ESE/ISE and ESS/ISS [2,6]. Such elements can be targeted by spliceogenic SRE-variants that may dysregulate splicing and be associated with a particular disease. The identification of active SREs is especially complex since they are usually degenerate sequences, and prediction tools generally present low accuracy. Alternatively, we have shown that SRE-functional mapping by overlapping microdeletions in splicing reporter minigenes is a valuable strategy to locate critical intervals for exon definition and potential clusters of spliceogenic SRE-variants. By means of this approach, we successfully mapped SREs in the breast cancer (BC) susceptibility genes *BRCA2* (MIM#600185), including exon 17 with a GC-5′ss, *CHEK2* (MIM#604373), and *RAD51D* (MIM#602954) [3,7,8,9]. Hence, *BRCA2* exon 17 was found to be regulated by several SR proteins, such as SC35 (SRSF2) or Tra2β, among others, that also intervene in the recognition of an anomalous GC site in the *BTK* gene [2,3].

In this study, we focused on GC-5′ss-exons of the breast and/or ovarian cancer (BOC) susceptibility genes [10,11,12,13,14], i.e., *ATM* (MIM#607585) exon 50, *BRIP1* (MIM#605882) exon 1, and *PALB2* (MIM#610355) exon 12, the last of which specifically undergoes skipping as a frequent alternative splicing event [15].

We formerly carried out three comprehensive studies of 114 splice site variants of *ATM* and *PALB2* [16,17,18] by RNA assays in seven minigenes. With two of these constructs (mgATM_49-52 and mgPALB2_5-12) and a new one of *BRIP1*, we proceeded to study the splicing regulation of these non-canonical splice sites by minigene assays. Several ESE-rich regions of *ATM* exon 50 and *PALB2* exon 12 were discovered, and 23 SRE-candidate and six control ± 1,2 variants were tested.

## 2. Materials and Methods

### 2.1. Variant and Transcript Annotations

Variants, transcripts, and predicted protein products were described according to the Human Genome Variation Society (HGVS) guidelines (https://varnomen.hgvs.org/), using the MANE selected transcripts Ensembl ENST00000675843.1 (Genbank NM_000051.4) for *ATM*, Ensembl ENST00000259008.7 (Genbank NM_032043.3) for *BRIP1* and Ensembl ENST00000261584.9 (Genbank NM_024675.4) for *PALB2*. We also annotated the transcripts according to a former shortened description.

### 2.2. Bioinformatics Analysis

Splice sites of the exons of interest were analyzed with the MaxEntScan (MES) algorithm (http://hollywood.mit.edu/burgelab/maxent/Xmaxentscan_scoreseq.html, accessed on 1 August 2021) [19].

*ATM* and *PALB2* variants were obtained from the ClinVar database (https://www.ncbi.nlm.nih.gov/clinvar/, accessed on 1 July 2021) [20] and analyzed with the HEXplorer software (https://rna.hhu.de/HEXplorer/, accessed on 1 July 2021) that calculates the splicing enhancer and silencer strengths of the sequences of interest and the presumed impact of variants on splicing [21]. Variants with scores < −40 were selected for subsequent RNA assays.

To identify the splicing factors involved in exon regulation, spliceogenic variants were analyzed with DeepCLIP (https://deepclip-web.compbio.sdu.dk/, accessed on 19 August 2024), a deep learning approach, which integrates RNA binding protein (RBP) functional data to predict the presence of splicing factor motifs [22]. This study was restricted to the known human SR proteins, hnRNPs, DAZAP1, TIA1, Tra2α, and Tra2β. Arbitrary cutoffs for DeepCLIP analysis were set as follows: RBP binding score > 0.8 for putative RBP binding site in the wild type (wt) and variant sequences, and a decrease in binding score by 0.3 or greater for binding site disruption.

The pathogenicity predictions of missense variants were performed by the deep learning approach AlphaMissense (https://alphamissense.hegelab.org/, accessed on 19 August 2024) [23] and the REVEL algorithm that integrates the scores of 13 individual prediction tools [24]. Both scores were calculated through the database dbNSFP v4.7a (academic version) (http://database.liulab.science/dbNSFP, accessed on 19 August 2024) developed for the functional prediction and annotation of non-synonymous single-nucleotide variants [25,26].

### 2.3. Minigenes

Two previously published minigenes, mgATM_49-52 and mgPALB2_5-12, as well as a new one of the *BRIP1* gene (mgBRIP1_1-2) were employed in this study [16,17]. All the constructs were validated in two different cell lines—MCF-7 and HeLa (mgATM_49-52) [16] and MCF-7 and MDA-MB-231 (mgBRIP1_1-2 and mgPALB2_5-12) [17]. A *BRIP1* insert with exon 2 and part of the flanking introns was cloned into the pSAD 9.0 vector using the restriction enzymes *Xba*I and *Bam*HI, yielding the minigene mgBRIP1_2. Exon 1 was cloned into the minigene mgBRIP1_2 by overlap extension PCR [27], replacing 62 bp of pSAD exon V1 and 363 nt of the vector intron and thus generating a chimeric exon V1–ex1 *BRIP1*. Finally, a 52 nt deletion was introduced into the 5′ end of exon 1 of *BRIP1* to eliminate an active cryptic 3′ss and stabilize the minigene (Supplementary Figure S1B).

**Figure 1:**
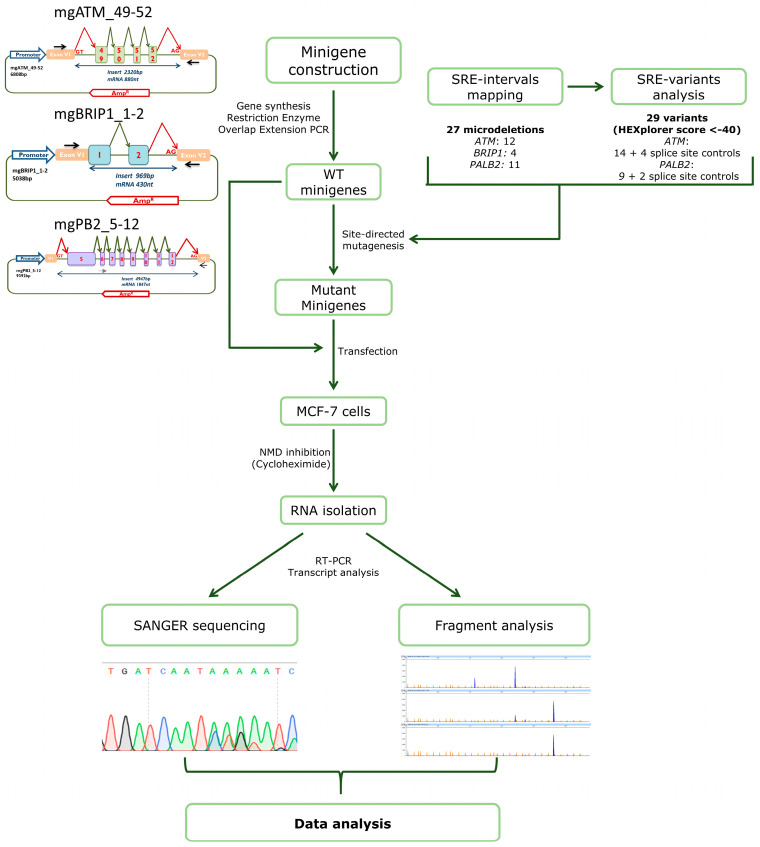
Workflow of the minigene assays. The basic assay includes the following steps: (1) Minigenes construction; (2) in silico analysis; (3) site-directed mutagenesis; (4) transfection of the wild type and mutant minigenes; (5) inhibition of nonsense-mediated decay and RNA purification; (6) transcript sequencing and fragment analysis by fluorescent capillary electrophoresis; (7) data interpretation.

The following seventeen microdeletions were introduced into the minigenes mgATM_49-52, mgBRIP1_1-2, and mgPALB2_5-12 (Figure 1; Supplementary Table S1) by site-directed mutagenesis: eight microdeletions in *ATM* exon 50 (c.7310_7339del, c.7335_7364del, c.7360_7389del, c.7385_7414del, c.7410_7439del, c.7435_7464del c.7460_7489del, and c.7485_7512del), four in *BRIP1* exons 1 and 2 (c.-88_-59del, c.-63_-34del, c.-28_1del, and c.-5_26del), and five in *PALB2* exon 12 (c.3204_3233del, c.3229_3258del, c.3254_3297del, c.3293_3322del, and c.3318_3347del). Thus, all the sequences of *ATM* exon 50 and *PALB2* exon 12 were covered by microdeletions. Additionally, 10 sub-microdeletions of *ATM* (c.7335_7344del, c.7343_7352del, c.7351_7360del, and c.7359_7368del) and *PALB2* (c.3229_3239del, c.3238_3248del, c.3247_3258del, c.3293_3303del, c.3302_3212del, and c.3311_3322del) were also introduced into the constructs.

Likewise, 14 *ATM* and 9 *PALB2* variants (HEXplorer scores < −40) were selected for subsequent RNA assays and genetically engineered into minigenes mgATM_49-52 and mgPALB2_5-12 (Supplementary Table S1). The wt minigenes were used as templates to generate all the mutant constructs with the QuikChange Lightning kit (Agilent, Santa Clara, CA, USA). All clones were confirmed by Sanger sequencing (Macrogen, Madrid, Spain).

### 2.4. Splicing Assays

Approximately 2 × 10^5^ MCF-7 cells were transfected with 1 µg of minigene using 2 µL of Lipofectamine LTX (Life Technologies, Carlsbad, CA, USA) as previously described. To inhibit nonsense-mediated decay (NMD), cells were incubated with cycloheximide 300 µg/mL (Sigma-Aldrich, St. Louis, MO, USA) for 4 h just before RNA extraction. RNA was purified with the Genematrix Universal RNA Purification Kit (EURx, Gdansk, Poland) including DNase I treatment.

Then, 400 ng of each sample RNA were retrotranscribed using the RevertAid First-Strand cDNA Synthesis Kit (Life Technologies) and the minigene exon V2-specific primer RTPSPL3-RV (5′-TGAGGAGTGAATTGGTCGAA-3′). Subsequently, cDNA (40 ng) was amplified using Platinum Taq polymerase (Life Technologies) and the following primer pairs:

- *ATM* and *BRIP1*: RTPSPL3-FW (5′-TCACCTGGACAACCTCAAAG-3′) and RTpSAD-RV (Patent P201231427).
- *PALB2*: RTPB2_EX6-FW (5′-GATAGCATAAACCCAGGCA-3′) and RTpSAD-RV.

After an initial denaturation at 94 °C/2 min, 35 PCR cycles were run as follows: 94 °C/30 s, 60 °C/30 s, and 72 °C (1 min/kb). Then, there was a final extension step at 72 °C/5 min. The expected minigene full-length (mgFL) transcripts were 880 (*ATM*), 430 (*BRIP1*) and 906 nt (*PALB2*).

To calculate the relative contribution of each transcript, semi-quantitative fluorescent RT-PCRs with a FAM-labelled oligonucleotide were conducted in triplicate under standard conditions, except that the number of cycles was 26. Fluorescent products were run with LIZ-600 (*BRIP1*) or 1200 (*ATM* and *PALB2*) Size Standards at the Macrogen facility (Seoul, Korea) and analyzed using Peak Scanner software V1.0 (Life Technologies). Only peak heights ≥ 50 RFU (Relative Fluorescence Units) were considered. The mean peak areas from three separate experiments for each variant were utilized to calculate both the relative proportions of each transcript and standard deviations. The whole procedure is outlined in Figure 1.

## 3. Results

A simple visual inspection of the GC-donor sites of the BOC susceptibility genes *ATM*, *BRIP1*, *PALB2*, and *BRCA2* shows that three of them (*BRIP1* exon 1, *PALB*2 exon 12, and *BRCA2* exon 17) are identical (CAGgcaagt), while *ATM* exon 50 only differs in the first position (A vs. C). They resemble the canonical 5′ splice site consensus sequence (MAGgXaagt). Their MES scores of around 3.0 indicate weak donor sites while their corresponding hypothetical GT sites’ scores would indicate strong 5′ss, as expected by their high sequence conservation as follows: *ATM*, AAGgcaagt (MES 3.24) vs. AAGgtaagt (11.00); *BRIP1*, *PALB2* and *BRCA2*: CAGgcaagt (MES 3.10) vs. CAGgtaagt (10.86).

### 3.1. Functional Mapping of Splicing Regulatory Elements

**Figure 2:**
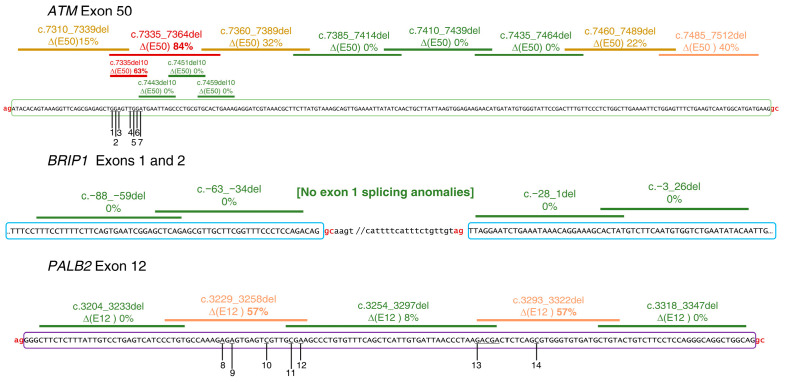
Map of microdeletions and tested variants of *ATM* exon 50, *BRIP1* exons 1 and 2, and *PALB2* exon 12. Exon sequences are in uppercase and boxed. Microdeletions are indicated by lines: green, weak or no impact on target exon recognition; yellow, moderate impact; orange, moderate to strong impact; red, strong impact. Variants are underlined: 1, c.7335G>W; 2, c.7336G>T; 3, c.7337A>K; 4, c.7340T>R; 5, c.7341G>A; 6,c.7342G>H; 7, c.7343A>B; 8, c.3242A>G; 9, c.3244A>G; 10, c.3251C>W; 11, c.3256C>G; 12, c.3258A>T; 13, c.3294_3298del; 14, c.3306C>K [Ambiguity codes: W=A/T; K=G/T; R=A/G; H=A/C/T; B=C/G/T].

The efficient inclusion of exons with atypical GC-donors may involve the participation of SREs as we have previously shown in the *BRCA2* exon 17 [3]. To locate active SREs, a total of 17 microdeletions (28–44 nt) were introduced into minigenes mgATM_49-52, mgBRIP1_1-2, and mgPALB2_5-12. These deletions covered the complete sequences of *ATM* exon 50 and *PALB2* exon 12, as well as 55 nt of the 3′ and 5′ ends of *BRIP1* exons 1 and 2, respectively. In all cases, the conserved positions of the splice sites (first two and three last exonic nt) were preserved in each exon. We found three intervals whose removal significantly affected exon definition, i.e., *ATM* c.7335_7364del (exon 50 skipping, ∆(E50) in >80% of transcripts), *PALB2* c.3229_3258del, and c.3293_3322del (exon 12 skipping, ∆(E12): 57%) (Figure 2; Supplementary Table S2). These data indicate that these intervals likely contain splicing enhancers. On the other hand, the four *BRIP1* microdeletions did not impair splicing, so we can conclude that the GC-donor of exon 1 is not controlled by, at least, the SREs placed within the deleted sequences.

We therefore proceeded to refine the SRE map with further internal sub-microdeletions of 10–12 nt. Only *ATM* interval c.7335_7344del significantly impaired exon 50 recognition (63% of transcripts), while the rest of the sub-microdeletions did not induce a remarkable impact.

### 3.2. Splicing Assays of Variants

Potential spliceogenic variants affecting SREs should be concentrated in the three presumed ESE-rich intervals (*ATM* c.7335_7344 and *PALB2* c.3229_3258del and c.3293_3322del) those segments. We employed the HEXplorer tool to select candidate variants with scores < −40 (previously used thresholds: <−5 and −20) with a view to increase its accuracy [7,28]. Thus, 14 *ATM* and 9 *PALB2* variants were selected and genetically engineered into their corresponding minigenes and tested in MCF-7 cells.

**Figure 3:**
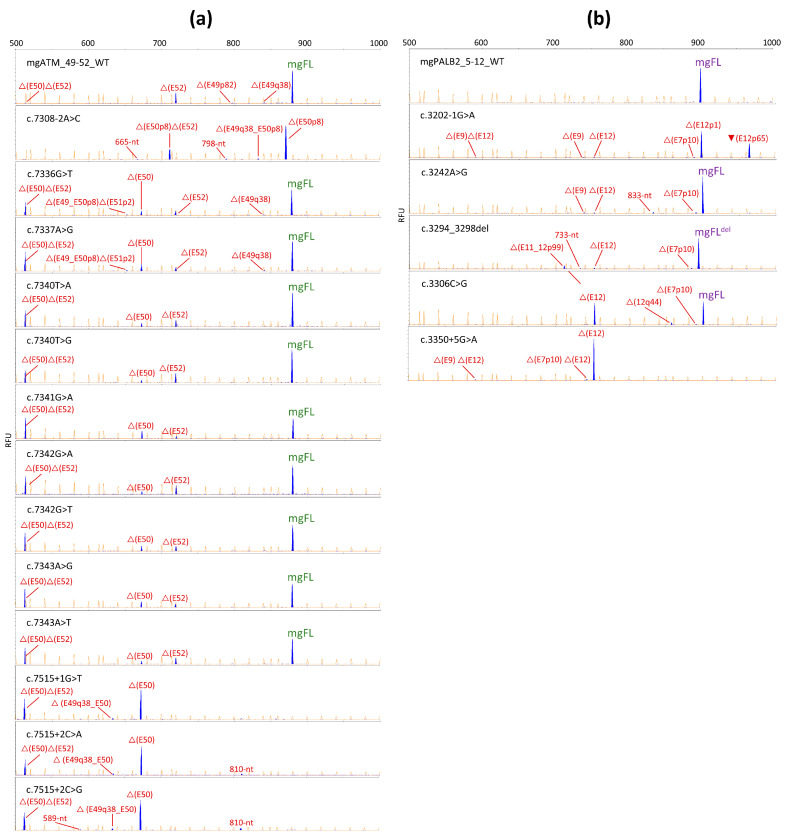
Splicing functional assays of spliceogenic variants. (**a**) *ATM* exon 50. (**b**) *PALB2* exon 12. Fluorescent fragment analysis of generated transcripts by wild-type, controls and mutant minigenes. FAM-labeled products (blue peaks) were run with LIZ-1200 (orange peaks) as size standard. mgFL, minigene full-length transcripts. Transcripts produced by c.3294_3298del contain a 5 nt deletion.

*ATM.* The wt minigene produces 73% of the mgFL-transcript and several alternative transcripts, as previously reported. So, we established 63% of the mgFL-transcript (10% reduction) as the cutoff for a variant to be considered spliceogenic. As expected, the control ± 1,2 variants c.7308-2A>C and c.7515 + 1G>T completely disrupted splicing (Table 1). Nine exonic variants (six predicted missense, two nonsense, and one synonymous variants) affected splicing, significantly increasing the transcripts with exon 50 skipping (23–54%) (Table 1, Figure 3a). Thus, these variants likely alter the SREs involved in exon 50 recognition. Interestingly, the most spliceogenic variant c.7341G>A (△(E50), 54%) is a synonymous change (p.Leu2447=), confirming that regardless of the predicted protein translation, any nucleotide change is capable of disrupting splicing as previously reported [29]. △(E50) transcripts (△(E50) and △(E50)△(E52)) introduce a premature termination codon (PTC) that are predicted to be degraded by the NMD process so that they can be considered loss-of-function isoforms.

*PALB2*. The wt mgPALB2_5-12 is known to produce 100% mgFL-transcript while, as expected, the control 5′ss and 3′ss variants completely disrupted splicing without any trace of the mgFL-transcript. Three variants reduced the expression of the mgFL-transcript by >10% so they were considered spliceogenic (Table 1, Figure 3b). Two variants (c.3242A>G and c.3294_3298del) had a weak impact (mgFL-transcript: 83–89%). Only c.3306C>G significantly impaired exon 12 recognition (exon 12 skipping, △(E12), 45% of the overall expression), highlighting that this variant disrupts critical SREs (Table 1, Figure 3b). △(E12) is a loss-of-function frameshift transcript with a PTC (p.(Gly1068Valfs*5)) affecting the folding WD40 domain, so it was previously established as a very strong signal of pathogenicity [17]. Finally, c.3306G>C also strengthens a cryptic 5′ss (MES: 1.59 → 5.37) that induced transcript △(E12q44) (4.7% of the overall expression). The other two spliceogenic variants (c.3242A>G and c.3294_3298del) barely impaired exon 12 inclusion (△(E12): 2.5% and 5.4%, respectively), so both were disregarded for subsequent analyses.

### 3.3. Analysis of + 2C>A,G Variants

There are other rare functional splice sites, such as the GG- and GA-5′ss which account for 0.01% and 0.006% of human exons, respectively [5]. Thus, we previously reported an active atypical GG-donor site at exon 12 of the *ATM* gene generated by the variant c.1898 + 2T>G that induced a 13% full-length transcript [16]. Then, we proceeded to check whether the *ATM* exon 50 GC-donor could tolerate + 2G, A substitutions and thus generating the functional donor sites GG and GA, respectively. Both variants, c.7515 + 2C>A and c.7515 + 2C>G, did not produce any trace of the mgFL-transcript, so the dinucleotides GG and GA did not work as functional 5′ss (Table 1, Figure 3a).

### 3.4. Splicing Factors

DeepClip analysis was carried out as indicated in Section 2.2. (Supplementary Table S3). Seven spliceogenic *ATM* variants (c.7336G>T, c.7337A>G, c.7341G>A, c.7342G>A, c.7342G>T, c.7343A>G, and c.7343A>T, exon 50 skipping: 33–54%) showed a marked decrease in the binding capacity of the SR proteins SRSF1 (SF2/ASF) and SRSF9. SRSF1 is an abundant and highly conserved protein involved in multiple processes of RNA metabolism, including the regulation of constitutive and alternative splicing, mRNA export, translation, etc. [30,31], as well as the recognition of GC-5′ss [2,3]. Other factors (e.g., DAZAP1 or SRSF2) showed changes in their binding capacity but a common pattern among variants could not be identified. Curiously, the most spliceogenic variant, c.7341G>A (△(E50) transcripts 54%), increased the binding score for the theoretical repressor proteins hnRNP A2B1 and L, which could explain its higher splicing impact.

In the case of *PALB2* c.3306C>G, a significant decrease in the binding score of SRSF2, SRSF7, hnRNP L, and TIA1 was observed. Interestingly, SRSF2 (SC35) and SRSF7 (9G8) had already been implicated in GC-5′ss recognition [2]. On the other hand, SRSF1 displayed contradictory results, as it showed losses and gains of binding capacity. Like *ATM* c.7341G>A, this variant concomitantly increased the binding probability for the repressor proteins hnRNP A1 and hnRNP A1L2 (0.95 and 0.84, respectively), such that a combined effect ESE disruption/ESS creation could be responsible for their greater splicing impact.

## 4. Discussion

The splicing process is a key gene expression step that is controlled by multiple factors, including splice site strength, genomic environment, RNA secondary structure, transcription kinetics and splicing enhancers and silencers [32]. Furthermore, it is expected that non-canonical splice sites, such as GC-5′ss, also require special conditions with the participation of several splicing factors, as previously reported [2,3]. In this study, we have found that inclusions of *ATM* exon 50 and *PALB2* exon 12 (both with atypical GC-5′ss) into the mature mRNA depend on the integrity of several exonic sequences. In this regard, SRE-mapping by microdeletion analysis in minigenes is a useful strategy for mapping SRE-rich intervals that can outperform any in silico prediction algorithm. Through this strategy, we have already mapped SREs in 13 *BRCA2* exons, *RAD51D* exon 3, *CHEK2* exons 8 and 10 and, in this study, *ATM* exon 50 and *PALB2* exon 12 [3,7,8,33]. Moreover, the minigene splicing assays of the variants provide valuable data for initial risk assessment, particularly if variant-carrier RNA is not available. Moreover, splicing reporter minigenes are versatile tools that virtually allow for the study of any disease gene (http://www.ibgm.med.uva.es/servicios/servicio-de-splicing-minigenes/, accessed on 1 October 2024) [34].

Hence, this approach has been proven to be very useful to assess the cryptic spliceogenic exonic variants affecting enhancer and/or silencer sequences. Thus, we identified nine *ATM* and three *PALB2* variants (52% of all tested variants) that disrupted the recognition of their corresponding exons, although none of them had a total impact. Yet, microdeletions, such as *ATM* c.7335_7364del (16.4% mgFL-transcript), provoked greater splicing impacts, suggesting that it should encompass several binding motifs. As we previously proposed, exon identification would be the result of the cooperation between several splicing factors, such that the elimination of a particular binding motif by a single-nucleotide variant may have a discrete effect on splicing [8,35]. In fact, DeepCLIP predictions suggest the participation of several splicing factors including SRSF1 and SRSF9 for *ATM* variants or SRSF2 and SRSF7 for *PALB2* c.3306C>G, among others.

With regard to the clinical interpretation of these variants, △(E50) and △(E12) are considered loss-of-function transcripts and therefore very strong signals of pathogenicity [16,17]. However, all the spliceogenic variants produce > 40% of their corresponding mgFL-transcripts. While the *PALB2* expression-cutoff for haplosufficiency is unknown, we previously showed that variants expressing ≥ 30% of the *ATM* full-length transcript cannot be classified as pathogenic [16] or, in other words, these variants would keep their tumor-suppressor activity. Therefore, in the absence of other pathogenic evidence (rarity, case-control studies, presence in ataxia telangiectasia patients in the case of *ATM*, etc.), these *ATM* variants cannot be considered loss-of-function/pathogenic variants based on splicing data alone. On the other hand, all the control ± 1,2 variants (*ATM* c.7308-2A>C, c.7515 + 1G>T, c.7515 + 2C>A and c.7515 + 2C>G, and *PALB2* c.3202-1G>A and c.3350 + 5G>A) induce complete aberrant splicing patterns with no traces of the mgFL-transcript. Moreover, they generated 91–100% PTC-NMD transcripts, so a priori they should probably be classified as pathogenic/likely pathogenic variants. Nevertheless, a comprehensive interpretation according to the current classification guidelines, such as those of the American College of Medical Genetics and Genomics and the Association for Molecular Pathology (ACMG/AMP), should be carried out. Thus, *PALB2* c.3350 + 5G>A was previously classified as pathogenic according to an ACMG/AMP variant classification scheme [17,36].

On the other hand, the spliceogenic *ATM* variants c.7336G>T and c.7340T>A express the mgFL-transcripts carrying premature termination codons (p.(Glu2446Ter) and p.(Leu2447Ter), respectively). As per the classification rules specified for *ATM* variants (https://www.clinicalgenome.org/affiliation/50039/, accessed on 1 October 2024), based on the ACMG/AMP guidelines [37], nonsense variants are considered very strong evidence of pathogenicity. Taken together, c.7336G>T and c.7340T>A could be classified as pathogenic, since both inactivate ATM by a double deleterious mechanism, splicing disruption and protein truncation (mgFL-transcript with PTC), which in turn might change disease penetrance and risk. Interestingly, homozygosity for certain *BRCA1* nonsense variants is viable due to the alternative splicing isoform ∆11q that partially retains BRCA1 function and skips the downstream exon 11 nonsense variants, causing a Fanconi-like syndrome [37].

Those spliceogenic variants producing mgFL-transcripts with a missense change are more complex to interpret. *ATM* variants c.7342G>A, c.7342G>T, c.7343A>G, and c.7343A>T at codon 2448 (p.Asp2448 to Asn, Tyr, Gly, and Val, respectively) and *PALB2* c.3306C>G (p.(Ser1102Arg)) were catalogued as likely pathogenic (*p* = 0.9–0.95 and 0.62, respectively; Supplementary Table S4) by the deep learning tool AlphaMissense [24]. REVEL analysis supported pathogenicity for ATM p.(Leu2447Trp), p.(Asp2448Tyr), p.(Asp2448Gly), and p.(Asp2448Val) but not for ATM p.(Asp2448Asn) and PALB2 p.(Ser1102Arg). Importantly, ATM-Asp2448 is located in the essential TRD3 domain involved in ATM dimerization and DNA-mediated activation [38]. PALB2-Ser1102 is one of residues in the WD40 5D β-strand (p.1101–1108) that is critical for folding of the WD40 domain [39]. However, experimental studies have shown that p.(Ser1102Arg) does not significantly impair PALB2 protein function [40]. Consequently, in silico predictions of missense pathogenicity are not accurate and should not be used as the unique evidence for variant classification [41]. Other forms of proof of pathogenicity, including protein function assays, are required to confirm their role in disease susceptibility. Remarkably, our study highlights the relevant contribution of predicted missense variations to anomalous splicing as a mechanism of disease susceptibility, as we have already shown in the other BC genes, such as *BRCA2* c.451G>A (p.(Val151Ile)) or *CHEK2* c.883G>A (p.(Glu295Lys)) and c.884A>T (p.(Glu295Val)) [8]. This splicing impact of predicted missense variants was also observed in other disease genes, like *CAPN3*, responsible for limb–girdle muscle dystrophy, in which 7 out of 21 missense variants disrupted splicing [42].

## 5. Conclusions

Splicing is a complex gene expression process that is controlled by a wide array of *cis*-acting elements. These motifs are particularly concentrated at certain exons, such as alternative or special exons with non-canonical splice sites, whose recognition and inclusion in the mature mRNA require the intervention of additional splicing factors (SR proteins and hnRNPs). We have shown that exonic variants at these motifs impair splicing and can potentially be associated with cancer risk.

Although splicing disruption by variants is usually underestimated, the mis-splicing of BC genes is a relatively frequent mechanism of gene inactivation [29]. In fact, we already performed the splicing assays of 706 variants of the BC susceptibility genes *BRCA1*, *BRCA2*, *CHEK2*, *RAD51C*, *RAD51D*, *PALB2*, and *ATM*, of which 431 (61%) induced splicing disruptions and 243 (34%) were classified as pathogenic or likely pathogenic. According to the ClinVar database, more than 25,000 variants of uncertain clinical significance have been described in the eight main BC predisposition genes, the clinical classification of which poses a challenge for medical genetics laboratories. To this end, experimental splicing data supply essential information for the interpretation of DNA variants and therefore for the clinical management of patients and families.

## Figures and Tables

**Table 1:** Splicing outcomes of selected variants at the spliceogenic intervals of *ATM* exon 50 and *PALB2* exon 12.

Variant ^1^	Hexplorer Score	Full-Length Transcript	Variant-Induced Transcripts
mgATM_49-52_WT		73.3% ± 0.4%	PTC-NMD: [△(E50)△(E52)] [1.3% ± 0.6%]; △(E49p82) [1.6%]; △(E49q38) [1.7% ± 0.2%] In-frame: △(E52) [22.1% ± 0.4%]
**c.7308-2A>C**	-	-	PTC-NMD: [△(E50p8)△(E52)] [23.8% ± 1.2%]; △(E49q38_E50p8) [1.7% ± 0.1%]; △(E50p8) [71.4% ± 1.6%]; Uncharacterized: 798-nt [1.6% ± 0.2%]; 665-nt [1.5% ± 0.1%]
c.7335G>A p.(Leu2445=)	−88.55	68.3% ± 1%	PTC-NMD: △(E50) [6% ± 0.1%]; [△(E50)△(E52)] [16.7% ± 0.6%]; △(E49q38) [1.4% ± 0.1%] In-frame: △(E52) [7.6% ± 0.4%]
c.7335G>T p.(Leu2445=)	−101	69.3% ± 0.2%	PTC-NMD: △(E50) [2.4%]; [△(E50)△(E52)] [8.4% ± 0.6%]; △(E49q38) [1.4% ± 0.1%]; [△(E49_E50p8)△(E51p2)] [2% ± 0.2%] In-frame: △(E52) [16.5% ± 0.3%]
**c.7336G>T** p.(Glu2446Ter)	−99.6	55.5% ± 0.6%	PTC-NMD: △(E50) [9.4% ± 0.2%]; [△(E50)△(E52)] [23.5% ± 0.5%]; △(E49q38) [1.3%]; [△(E49_E50p8)△(E51p2)] [1.4% ± 0.6%] In-frame: △(E52) [8.9% ± 0.1%]
**c.7337A>G** p.(Glu2446Gly)	−153.63	50.8% ± 0.7%	PTC-NMD: △(E50) [11.8%]; [△(E50)△(E52)] [28.1% ± 0.3%]; △(E49q38) [1.2% ± 0.1%]; [△(E49_E50p8)△(E51p2)] [1.5% ± 0.1%] In-frame: △(E52) [6.6% ± 0.2%]
c.7337A>T p.(Glu2446Val)	−42.76	68.4% ± 0.7%	PTC-NMD: [△(E50)△(E52)] [3.5% ± 0.1%]; △(E49q38) [1.6% ± 0.1%]; △(E49p82) [1.2%]; [△(E49_E50p8)△(E51p2)] [2% ± 0.1%] In-frame: △(E52) [23.3% ± 0.4%]
**c.7340T>A** p.(Leu2447Ter)	−57.36	58.8% ± 0.7%	PTC-NMD: [△(E50)△(E52)] [22.7% ± 1.6%]; △(E50) [6.3% ± 0.2%] In-frame: △(E52) [12.2% ± 1.4%]
**c.7340T>G** p.(Leu2447Trp)	−71.76	61.8% ± 1.5%	PTC-NMD: [△(E50)△(E52)] [18.6% ± 1.3%]; △(E50) [4.3% ± 0.2%] In-frame: △(E52) [15.3% ± 1.5%]
**c.7341G>A** p.(Leu2447=)	−52.54	40.4% ± 0.5%	PTC-NMD: [△(E50)△(E52)] [38.4% ± 0.8%]; △(E50) [16% ± 0.2%] In-frame: △(E52) [5.2% ± 0.3%]
**c.7342G>A** p.(Asp2448Asn)	−66.8	50.2% ± 0.8%	PTC-NMD: [△(E50)△(E52)] [27.9% ± 1%]; △(E50) [5.2% ± 0.1%] In-frame: △(E52) [16.7% ± 0.6%]
c.7342G>C p.(Asp2448His)	−68.24	63.9% ± 0.1%	PTC-NMD: [△(E50)△(E52)] [16.9% ± 0.3%]; △(E50) [4.5% ± 0.1%] In-frame: △(E52) [14.7% ± 0.2%]
**c.7342G>T** p.(Asp2448Tyr)	−95.7	51.3% ± 0.8%	PTC-NMD: [△(E50)△(E52)] [29.9% ± 0.7%]; △(E50) [9.6% ± 0.1%] In-frame: △(E52) [9.2%]
c.7343A>C p.(Asp2448Ala)	−43.86	73.8% ± 1%	PTC-NMD: [△(E50)△(E52)] [6.6% ± 0.1%] In-frame: △(E52) [19.6% ± 0.9%]
**c.7343A>G** p.(Asp2448Gly)	−111.28	49.4% ± 1.2%	PTC-NMD: [△(E50)△(E52)] [31.9% ± 0.7%]; △(E50) [11.7% ± 0.2%] In-frame: △(E52) [7% ± 0.3%]
**c.7343A>T** p.(Asp2448Val)	−96.18	52.6% ± 0.4%	PTC-NMD: [△(E50)△(E52)] [28.9% ± 0.4]; △(E50) [6.1% ± 0.2%] In-frame: △(E52) [12.4% ± 0.1%]
**c.7515 + 1G>T**	-	-	PTC-NMD: △(E50) [58.7% ± 1.2%]; [△(E50)△(E52)] [38.5% ± 1.2%] In-frame: △(E49q38_E50) [2.8% ± 0.1%]
**c.7515 + 2C>A**	-	-	PTC-NMD: △(E50) [62.5% ± 1.3%]; [△(E50)△(E52)] [32.3% ± 0.5%] In-frame: △(E49q38_E50) [3.4% ± 0.1%] Uncharacterized: 810-nt [1.8% ± 1.8%]
**c.7515 + 2C>G**	-	-	PTC-NMD: △(E50) [59.6% ± 1.5%]; [△(E50)△(E52)] [31.1% ± 0.5%] In-frame: △(E49q38_E50) [3.2% ± 0.1%] Uncharacterized: 474-nt [1.4%]; 589-nt [1.2% ± 0.1%]; 810-nt [3.5% ± 1.3%]
mgPALB2_5-12_WT		100%	
**c.3202-1G>A**	-	-	PTC-NMD: △(E12p1) [60.1% ± 0.3%]; ▼(E12p65) [33.4% ± 0.6%]; △(E7p10) [1.8% ± 0.1]; △(E12) [1.8% ± 0.5%]; [△(E9)△(E12)] [1.3% ± 0.1%] In-frame: △(E9) [1.6% ± 0.1%]
**c.3242A>G** p.(Glu1081Gly)	−67.77	89.7% ± 0.8%	PTC-NMD: △(E12) [2.5% ± 0.5%]; △(E7p10) [2.3% ± 0.2%] In-frame: △(E9) [2.4% ± 0.3%] Uncharacterized: 833-nt [3.1% ± 0.4%]
c.3244A>G p.(Ser1082Gly)	−65.81	94.7% ± 0.4%	PTC-NMD: △(E7p10) [2.5% ± 0.5%]; [△(E7p10_△(E11_12] [1.5% ± 0.1%] In-frame: △(E9) [1.3% ± 0.1%]
c.3251C>T p.(Ser1084Leu)	−45.61	96.9% ± 0.3%	PTC-NMD: △(E7p10) [3.1% ± 0.3%]
c.3251C>A p.(Ser1084Ter)	−50.22	95.4% ± 0.3%	PTC-NMD: △(E7p10) [3.4% ± 0.3%] In-frame: △(E9) [1.2% ± 0.1%]
c.3256C>G p.(Arg1086Gly)	−48.73	94.2% ± 0.5%	PTC-NMD: △(E7p10) [3.3% ± 0.5%]; △(E12 [1% ± 0.1%] In-frame: △(E9) [1.5% ± 0.1%]
c.3258A>T p.(Arg1086=)	−57.31	94.3% ± 0.5%	PTC-NMD: △(E7p10) [2.5% ± 0.5%]; △(E12) [1.7% ± 0.1%] In-frame: △(E9) [1.5%]
**c.3294_3298del** p.(Lys1098Asnfs*23)	−63.22	83.1% ± 1.4%	PTC-NMD: [△(E11_12p99)] [7.3% ± 3.5%] △(E12) [5.4% ± 3.2%]; △(E7p10) [2.6% ± 0.2%] Uncharacterized: 733-nt [1.6% ± 0.6%]
**c.3306C>G p.(Ser1102Arg)**	−143.8	49.3% ± 1.6%	PTC-NMD: △(E12) [44.6% ± 1.8%]; △(E12q44) [4.7% ± 0.1%]; △(E7p10) [1.4% ± 0.1%]
c.3306C>T p.(Ser1102=)	−53.46	92.9% ± 0.4%	PTC-NMD: △(E12) [3% ± 0.1%]; △(E7p10) [2.8% ± 0.2%] In-frame:△(E9) [1.3% ± 0.1%]
**c.3350 + 5G>A ^2^**	-	-	PTC-NMD: △(E12) [100%]

^1^ Spliceogenic Variants (*ATM* mgFL < 63% and *PALB*2 mgFL < 90%) are shown in bold. ^2^ Valenzuela-Palomo et al. (2022) [17].

## Data Availability

Sequencing and fragment analysis data are available at http://hdl.handle.net/10261/367824, accessed on 20 October 2024 (https://doi.org/10.20350/digitalCSIC/16556).

## Supplementary Materials

The following supporting information can be downloaded at: https://www.mdpi.com/article/10.3390/cancers16213562/s1, Figure S1: Insert sequences of minigenes mgATM_49-52, mgBRIP1_1-2 and mgPALB2_5-12; Table S1: BRIP1 cloning primers and mutagenesis primers for microdeletions and variants; Table S2: Splicing outcomes of ATM, BRIP1 and PALB2 microdeletions; Table S3: DeepCLIP analysis of ATM and PALB2 spliceogenic variants; Table S4: AlphaMissense and REVEL scores of ATM and PALB2 spliceogenic missense variants.
