# Supplementary material for: Splicing Dysregulation of Non-Canonical GC-5′ Splice Sites of Breast Cancer Susceptibility Genes ATM and PALB2
Source: Cancers (Basel). 2024 Oct 22;16(21):3562. doi: 10.3390/cancers16213562 (PMC11545216; doi:10.3390/cancers16213562)
Supplement: Supplementary file 1 [file cancers-16-03562-s001.zip › Supplementary_Material_R1.pdf]

Supplementary Figure S1. Insert sequences of minigenes mgATM_49-52, mgBRIP1_1-2 and mgPALB2_5-12. Exons are coloured and in upper case and cloning sites are underlined. The structure of each insert is indicated below each sequence, where a double slash indicates a shortened intron.

S1-A. Insert sequence of minigene mgATM_49-52
S1-B. Insert sequence of minigene mgBRIP1_1-2
S1-C. Insert sequence of minigene mgPALB2_5-12

RE: *Bam*HI

GGATCCaaaatcacaaaagaaatctcataatgttttaagaaaatgtacgaatttgtgttgggccacattcaaagccgtcctggg
ccacatgcggcccatgggccgtgggttggacaagtttgcaatagttcatataatttagctagcttttatatgtatataagttaaatttta
gtgtattaccttaatttgagtgattctttagatgtatttagtatttgtaaatataatttaaattggttgtgttttcttgaag**GCAGTAGAA**
GTTGCTGGAAATTATGATGGAGAAAGTAGTGATGAGCTAAGAAATGGAAAAATGAAGGCATTTCT **Ex49**
CTCATTAGCCCGGTTTTCAGATACTCAATACCAAAGAATTGAAAACTACATGAAATCATCGGAAT
TTGAAAACAAGCAAGCTCTCCTGAAAAGAGCCAAAGAGGAAGTAGGTCTCCTTAGGGAACATA
AAATTCAGACAAACAGgtaactaggtttctacaagtgacaattttatgttcaccagttaactgagtgagtgtttttgcatagaa
agagtgacttggtctttttatctgatatagttttgagctctaaaggtcggcttaactatatatagattatcttggtcttttgggttcttttcgg
tttttgttttttgttttttttttttgagacaaggtctcatcctttgatgcttaggaaggtgtgtgaattgcacagttaagacaaaagtaagttta
ttccctttataatccttagaagtttgctttttttccctgggataaaaacccaactttttttcattaaatgttgtatatcatgtgtgattttgtagt
tctgttaaagttcatggcttttgtgttttaccttaattattctatgcaag**ATACACAGTAAAGGTTCAGCGAGAGCTGG** **Ex50**
AGTTGGATGAATTAGCCCTGCGTGCACTGAAAGAGGATCGTAAACGCTTCTTATGTAAAGCAGT
TGAAAATTATATCAACTGCTTATTAAGTGGAGAAGAACATGATATGTGGGTATTCCGACTTTGTTC
CCTCTGGCTTGAAAATTCTGGAGTTTCTGAAGTCAATGGCATGATGAAGgcaagtgttactcagcccaat
attctaccctgtgcttgaaaaacttagacataagccccttgatgtcaggaatcgtgtatacctctttgtattcctagcacttggtccag
tgctctacacataagtagcattttgtagttttctaaactttgatccatatttaggattatttacaagttctagtcttgtttctacaaaagta
ccaatgcattaatctagagtacccattagaaagaccttcagataagaaaagaaatgaaggaaaacaatatagttagtgaagtttt
gttaaccacttgtgctaatagaggagcactgtcttaaaataacttacttgcttagatgtgagaatatttgaaataccttgtttcttaattt **Ex51**
tgtgtcttttttttaatggtag**AGAGACGGAATGAAGATTCCAACATATAAATTTTTGCCTCTTATGTACCAA**
TTGGCTGCTAGAATGGGGACCAAGATGATGGGAGGCCTAGGATTTCATGAAGTCCTCAATAATg
taagtaaacctgaaaatcaaaccacaataattatttttattctattattactatatattatataaagtatatataccattccctctaag
aaatggaaatacaaaattttgtattttttgtcttctcacatcacataagttactcattttctctctctaattcctcataggcctctgccttt
ttctcacacatgcaggcatacacgctctacccactgcagtatctagacagtaatacacattttaatgttaagcaaaatgaaaaat **Ex52**
atggattatattttttttgtttatttgcataaatctaatagttcttttcttacag**CTAATCTCTAGAATTTCAATGGATCACCC**
CCATCACACTTTGTTTATTATACTGGCCTTAGCAAATGCAAACAGAGATGAATTTCTGACTAAAC
CAGAGGTAGCCAGAAGAAGCAGAATAACTAAAAATGTGCCTAAACAAAGCTCTCAGCTTGATG
AGgtatttggattaaacatacgtaccttttagaagtgtgatattcagtctttcctagaatatttctttttaaaatcttgtgttattaagatg
ccatctaaaatcggttcaaggctggcacggtggctcacgcctgtaatcccagcactttgggaggctgaggcgggtggattacttga
ggtcagaagttcgagaccatcctggctgaccgacacagcaaaaccctgtctctactaaaaatgcaaaaaacagc**GAATTC**

RE: *Eco*R1

Supplementary Figure S1-A. Insert sequence of minigene mgATM_49-52. Structure of the insert (2,320 bp): ivs48 (250 bp) – ex49 (218 bp) – ivs49-1 (200 bp) // ivs49-2 (200 bp) – ex50 (208 bp) – ivs50-1 (200 bp) // ivs50-2 (200 bp) – ex51 (114 bp) – ivs51 (321 bp) – ex52 (159 bp) – ivs52 (250 bp)

A)

RE: *Xba*I

TCTAGAtgatttataagactaggaaattattttgtttccatttgttttgtttatacttttattaattccagtttctgtttccagatttctcccttagttttg
gttctctggttttctttgtaaggcgtgtctcaatatttgacattttcatttctgttgtagTTAGGAATCTGAAATAAACAGGAAAGCACTA
TGTCTTCAATGTGGTCTGAATATACAATTGGTGGGGTGAAGATTTACTTTCCTTATAAAGCTTACCCGTCACAG **Ex2**
CTTGCTATGATGAATTCTgtaagtatttttcagcagttaagtttttattaagatatagatacttatgacccataaagtacattgagtatttga
ctgcattcatattctcagtaaatggagaatattttctgggttcacttggaagaatatctaacctctgggaaactactgattgcagctaaactgtat
atatctgtggttaaggcaaagaagaacattctgaaaaatgttctcagctcatttggaatttctatacttttaatgaaaaac**GGATCC**
RE: *Bam*HI

B)

GGAATTCGTCTCGGGTTGTGTGGTTGAGGGGTCTGGTGGGTCGAGGAAAGGTAACGGCGGCCCCAGTCC
TGCACACAAGGCCGGGGAAGTAGCAGCACCCCCAGGAAGAGGGAGGAGGAAGGGCTCGTGCCCTTTCT **Ex1**
TCTCTTCCAGGGCTCCGCTTTATTTGCTCTCAGAAGTCGGTTTCCTTTCCTTTTCTTCAGTGAATCGGAGCTCA
GAGCGTTGCTTCGGTTTCCCTCCAGACAGgcaagtcggagggaagtccacggaaagggcttgagttcttctgcacagcctgcg
ggcccatgggtggtgactggatcaccagggtctcggggatctcaactggctgggggaggagggagatacggggcagaaaacctgaggaggg
agcctatccttacctcctttgagcgcaggcctgttcttagcccgggcgctgcgttaattcttggcgggaggcagaggggtgtgtttatcctttatgc
aatgtttgtgatttataagactaggaaattattttgtttccatttgttttgtttatacttttattaattccagtttctgtttccagatttctcccttagttttgg **Ex2**
ttctctggttttctttgtaaggcgtgtctcaatatttgacattttcatttctgttgtagTTAGGAATCTGAAATAAACAGGAAAGCACTAT
GTCTTCAATGTGGTCTGAATATACAATTGGTGGGGTGAAGATTTACTTTCCTTATAAAGCTTACCCGTCACAGC
TTGCTATGATGAATTCTgtaagtatttttcagcagttaagtttttattaagatatagatacttatgacccataaagtacattgagtatttgact
gcattcatattctcagtaaatggagaatattttctgggttcacttggaagaatatctaacctctgggaaactactgattgcagctaaactgtatat
atctgtggttaaggcaaagaagaacattctgaaaaatgttctcagctcatttggaatttctatacttttaatgaaaaac**GGATCC**
RE: *Bam*HI

C)

AACGGCGGCCCCAGTCCTGCACACAAGGCCGGGGAAGTAGCAGCACCCCCAGGAAGAGGGAGGAGG **Ex1**
AAGGGCTCGTGCCCTTTCTTCTCTTCCAGGGCTCCGCTTTATTTGCTCTCAGAAGTCGGTTTCCTTTCCTTTT
CTTCAGTGAATCGGAGCTCAGAGCGTTGCTTCGGTTTCCCTCCAGACAGgcaagtcggagggaagtccacggaaag
ggcttgagttcttctgcacagcctgcgggcccatgggtggtgactggatcaccagggtctcggggatctcaactggctgggggaggagggagat
acggggcagaaaacctgaggagggagcctatccttacctcctttgagcgcaggcctgttcttagcccgggcgctgcgttaattcttggcggga
ggcagaggggtgtgtttatcctttatgcaatgtttgtgatttataagactaggaaattattttgtttccatttgttttgtttatacttttattaattccagttt
ctgtttccagatttctcccttagttttggttctctggttttctttgtaaggcgtgtctcaatatttgacattttcatttctgttgtagTTAGGAATCTGA
AATAAACAGGAAAGCACTATGTCTTCAATGTGGTCTGAATATACAATTGGTGGGGTGAAGATTTACTTTCCTTA **Ex2**
TAAAGCTTACCCGTCACAGCTTGCTATGATGAATTCTgtaagtatttttcagcagttaagtttttattaagatatagatacttatga
cccataaagtacattgagtatttgactgcattcatattctcagtaaatggagaatattttctgggttcacttggaagaatatctaacctctgggaa
actactgattgcagctaaactgtatatatctgtggttaaggcaaagaagaacattctgaaaaatgttctcagctcatttggaatttctatactttta
atgaaaaac**GGATCC**
RE: *Bam*HI

Figure S1-B. Insert sequence of the *BRIP1* minigenes.

A) Structure of the insert included in mgBRIP1_2 (543 bp): ivs1 (156 bp) – Ex2 (123 bp) – ivs2 (252 bp)
B) Structure of the insert included in mgBRIP1_1-2 (1,027 bp): Ex1 (240 bp) – ivs1-1 (250 bp) // ivs1-2 (156 bp) – Ex2 (123 bp) – ivs2 (252 bp)
C) Structure of the insert included in mgBRIP1_1-2 stable (1,975 bp): Ex1 (188 bp) – ivs1-1 (250 bp) // ivs1-2 (156 bp) – Ex2 (123 bp) – ivs2 (252 bp)

agtcatggatgggaaaagtaatgaacatttttagtatatttgagtttagaagctcactctttgttgggtattacatttaagaatggtttaacatgtttcttg
ataggacttcattgtaaacattaagttcattctggggaaattaaggttcattaaaatgtttcttttaaatctaggagatcctattctctttgtcatcagtga
aacagattgtctgttttgttgggttttgttactattttgtgacttattttcttctttag**GGAAGAAAAGTCGTCATCAAAAAGAGGATTCCC**
TTTCTTGGAGTAATAGTGCTTATTTATCCTTGGATGATGATGCTTTCACGGCTCCATTTCATAGGGATGGAATG
CTGAGTTTAAAGCAACTACTGTCTTTTCTCAGTATCACAGACTTTCAGTTACCTGATGAAGACTTTGGACCTCT
TAAGCTTGAAAAAGTGAAGTCCTGCTCAGAAAAACCAGTGGAGCCCTTTGAGTCAAAAATGTTTGGAGAGAG
ACATCTTAAAGAGGGAAGCTGTATTTTTCCAGAGGAACTGAGTCCTAAACGCATGGATACAGAAATGGAGGA **Ex5**
CTTAGAAGAGGACCTTATTGTTCTACCAGGAAAATCACATCCCAAAAGGCCAAACTCGCAAAGCCAGCATA
CAAAGACGGGCCTTTCTTCATCCATATTACTTTATACTCCTTTAAATACGGTTGCGCCTGATGATAATGACAG
GCCTACCACAGACATGTGTTCACCTGCTTTCCCCATCTTAGGTACTACTCCAGCCTTTGGCCCTCAAGGCT
CCTATGAAAAAGCATCTACAGAAGTTGCTGGACGAACTTGCTGCACACCCCAACTTGCTCATTTGAAAGAC
TCAGTCTGTCTTGCCAGTGATACTAAACAATTCGACAGTTCAGGCAGCCCAGCAAAACCACATACCACCCT
GCAAGTGTCAGGCAGGCAAGGACAACCTACCTGTGACTGTGACTCTGTCCCGCCAGGAACACCTCCACC
CATTGAGTCATTCACTTTTAAAGAAAATCAGCTCTGTAGAAACACATGCCAGGAGCTGCATAAACATTCCGT
CGAACAGgtacaatccatttcctctgtgaaattttctctgaaggaatgaaatgccttagtgaatgtaaacagcatgacttgcttgcgcattgggcc
ttccacgtttaagaatggtttgacgtgtttctttgatatgatttcattgtaatcattaagttcattctggggaaattaaggttcattaaaatgtttcttttaaat
atgggaggtcctattctctttgttatcagtgaaacagtttgcatttggagctttgctgctgttataagaggaaataaagacaatacgaagtagacatttt
gatgagtgggtaatgcaggcagacattatacataaagtgtagactaatgatgtgacttttgttttcacag**ACTGAAACAGCAGAGCTTCCT** **Ex6**
GCTTCTGATAGCATAAACCCAGGCAACCTACAATTGGTTTCAGAGTTAAAGgtcagaagaatattctcttccagtgtctc
gtgtcttacatatgaaaactttaatgaactgaaaagaattcagtcatatagcttcttgttctttaatactattaaagatattggtaaacagattcagaaa
aacagatttggattggttatttttcccaatatttacctctgtttatgttttgagctctcctcttaaagtttctatgccaatgcaggaattcgatatcaagctt
atcgatacatgatgtaagtttgataggcatttttttaggtacataagaaccacaaagctctttcttttcacctgcataagacttaaattttacataccta
ctgtttcattgaattataattcatcactttgcatacttatgctttgcataaaacagcactcgagtgccactttaacagaactgttgccattgtgtcag**AA**
TCCTTCAGGTTCCTGTTCCGTAGATGTGAGTGCCATGTTTTGGGAAAGAGCCGGTTGTAAAGAGCCATGTAT **Ex7**
CATAACTGCTTGCGAAGATGTAGTTTCTCTTTGGAAAGCTCTGGATGCTTGGCAGTGGGAAAAACTTTATACC
TGGCACTTCGCAGAGgtaagtgggaatctcgagctgaaagagatctttgcagccatttgcctgataatgtagatgggcagcttaccaaaatt
gggagctatgaccatgcaaggcagaacagagatgaggcgtcgacatacccaattattcccctattgttagaattataccttgcattaggtaaatgct
cagtaagcactattatgctattatgcatatagtttatttagatttacagctaataaaaagagttttctgagccttcaaatgatgaaaattatccttgtaca
gtgagaatacaaaagaatgtgataaattttggaaaatctggattaaacaaaaatgaaacaaccaagcataatttttggctgctttgttttatttag**GT** **Ex8**
TCCAGTATTACAGATAGTTCCAGTGCCTGATGTGTATAATCTCGTGTGTGTAGCTTTGGGAAATTTGGAAATCA
GAGAGATCAGgtatgtaattcccaaggagtgatttgtttttccttcatctttgtctctgtcagctggttttaagtgcaggtaataacctaggcttgagt
cttgaaagaatctgaaagatctaaagagagagagatttgtttaaaaaaaaatcaatagaatgacatccctgactgaagtttctatttaaaatgtgaa
cctaggctgggcgcctcaggaggctgaggcaggacagtcgcttgaacctgggaggcagagttgcagtgagccaagattgtgccactgcactccag
cctaggcgacagagcaagactctgtctcaaaaaaaaaaaaaaaaaaaaagtgaacctagtcctttaatattaaaaggttactcctcacatcacc
ccatttttccttatatttggcttag**GGCATTGTTTTGTTCCTCTGATGATGAAAGTGAAAAGCAAGTACTACTGAAGTCTG** **Ex9**
GAAATATAAAAGCTGTGCTTGGCCTGACAAAGAGGAGGCTAGTTAGTAGCAGTGGGACCCTTTCTGATCAA
CAAGTAGAAGTCATGACGTTTGCAGAAGATGGAGGgtaagaaaagcattgattgatttttaactattagatgaagaatgattttatc
acaggtttcagagaaagttgggtaactaggatctcgtttttctgtgctgggggtgtaatataagcatgtaccgcatcaacactaggttatgacatagaa
gcaggttagtgaggtggaagccagacatgtcagggatgaagtcaaagaatgcttgcagcatatctaggtagatattatgaaagtaatatatgttcatt
gtagaaagtttagtatacacgttttctgggttagatttttttttcctgatattaggttagtttatattatgcagttcaacaatgcggagaagggctacctaga
gactgctttagtgcaaagtactgacttttcatactgttttaattacag**AGGCAAAGAAAACCAATTTTTGATGCCCCCTGAGGAGA** **Ex10**
CTATACTAACTTTTGCTGAGGTCCAAGGGATGCAAGAAGCTCTGCTTGGTACTACTATTATGAACAACATTGT
TATTTGgtaagctttccctctaggtcctcagttccctcatctgtagtatgaggatatacctctaattttacagggttgttgtgaagattaaataagaga
gtatgtgtaaacatgattgtggttttgtgttgctgttgttgttgtttttgttgtgttttgagacagagtctcctcctatcacccaggtgggagtacagtggtatg
atgctgaactatcaaatgaaactattggcaaaattaacccacagttctacttttacctaaatctatgactaaagaaaactaaggagctcttagtttttt
ccctggtcacctcctaagacatgctatgatgaataagaaaatattttctgaatactggtttgttggaagaatgtgatcagcttatttattttttgttatctaa
g**GAATTTAAAAACTGGTCAACTCCTGAAAAAGATGCACATTGATGATTCTTACCAAGCTTCAGTCTGTCACAA**
AGCCTATTCTGAAATGgtaagtaatgactggctgggaccactttgtgtgttactgttggtctcattaagtgagagcagtaagtcataagcagta **Ex11**
atgaacaaaccttccccgtggcatcttttttcaaaattggatagcaatgtttgtcctttcttgaaatttaggatgaaaataagtgttggtacagtgattgc
ttggtaaattttctggctgtaagccagaagtccagtccagtaataatctcaacagttcctagacggcagggaaaaaaaatcaagccagtggttaaat
cctggatacttcagagcctatcggtcattgctttaattgtttggtttttgtctctgccagatctttatttttcctgacatactcttgacagtctatttgggatat
ttatttttctccgaaattag**GGGCTTCTCTTTATTGTCCTGAGTCATCCCTGTGCCAAAGAGAGTGAGTCGTTGCGAA** **Ex12**
GCCCTGTGTTTCAGCTCATTGTGATTAACCCTAAGACGACTCTCAGCGTGGGTGTGATGCTGTACTGTCTTC
CTCCAGGGCAGGCTGGCAGgcaagtgtgcataactgctactctatgggtgggacattctgaaaggcactgtgcaaacacaaaaacta
gatatttttgtgtcacttagaagaatggaaaacatgaaatactatatatcatgataggaatattcagaagtaggatgaagattagtggacttgagtcaa
accccagattccctgcatcctgatctctactagtatggggttatataggtttttaatagtgtttattcaagccctgtttttttc**GGTACC**
RE: *Kpn*I

Figure S1-C. Insert sequence of minigene mgPALB2_5-12. Structure of the insert (4,947 bp): ivs4 (266 bp) – ex5 (830 bp) – ivs5 (364 bp) – ex6 (72 bp) – ivs6 (209 bp) // ivs6 (191 bp) – ex 7 (162 bp) – ivs7 (114 bp) // ivs7 (250 bp) – ex 8 (86 bp) – ivs8 (200 bp) // ivs8 (200 bp) – ex9 (162 bp) – ivs9 (200 bp) // ivs9 (200 bp) – ex 10 (117 bp) – ivs10 (200 bp) // ivs10 (200 bp) – ex11 (88 bp) – ivs11-1 (200 bp) // ivs11-2 (200 bp) – ex 12 (149 bp) – ivs12 (250 bp)

Supplementary Table S1. *BRIP1* cloning primers and mutagenesis primers for microdeletions and variants.

mgBRIP1_1-2 minigene cloning primers	
**Primers cloning**	**Primer (5’ →3’)**
BRIP1_ex2_XBAI_FW	TATATATCTAGATGATTTATAAGACTAGGAAATTATT
BRIP1_ex2_ BAMHI _RV	TATATAGGATCCGTTTTTCATTAAAAGTATAGAAATT
BRIP1_ex1_FW	GGTCTGAGTCACCTGGACAACCTCAAAGGCGGAATTCGTCTCGGGTTGTG
BRIP1_ex1 _RV	AACAAAATAATTTCCTAGTCTTATAAATCACAAACATTGCATAAAGGATAAACAC
BRIP1_delex1_52pb	GAGTCACCTGGACAACCTCAAAGGCAACGGCGGCCCCAGTCCTGCACACA<br>TGTGTGCAGGACTGGGGCCGCCGTTGCCTTTGAGGTTGTCCAGGTGACTC

Microdeletion	Gene	Primers (5’ →3’)
c.7310_7339del	*ATM*	GTGTTTTACCTTAATTATTCTATGCAAGATTGGATGAATTAGCCCTGCGTGCACTGAAAG<br>CTTTCAGTGCACGCAGGGCTAATTCATCCAATCTTGCATAGAATAATTAAGGTAAAACAC
c.7335_7364del		AAGATACACAGTAAAGGTTCAGCGAGAGCTGAAAGAGGATCGTAAACGCTTCTTATGTAA<br>AAGATACACAGTAAAGGTTCAGCGAGAGCTGAAAGAGGATCGTAAACGCTTCTTATGTAA
c.7360_7389del		GAGCTGGAGTTGGATGAATTAGCCCTGCGTTGTAAAGCAGTTGAAAATTATATCAACTGC<br>GCAGTTGATATAATTTTCAACTGCTTTACAACGCAGGGCTAATTCATCCAACTCCAGCTC
c.7385_7414del		TGCGTGCACTGAAAGAGGATCGTAAACGCTACTGCTTATTAAGTGGAGAAGAACATGATA<br>TATCATGTTCTTCTCCACTTAATAAGCAGTAGCGTTTACGATCCTCTTTCAGTGCACGCA
c.7410_7439del		ACGCTTCTTATGTAAAGCAGTTGAAAATTATGATATGTGGGTATTCCGACTTTGTTCCCT<br>AGGGAACAAAGTCGGAATACCCACATATCATAATTTTCAACTGCTTTACATAAGAAGCGT
c.7435_7464del		AATTATATCAACTGCTTATTAAGTGGAGAATCCCTCTGGCTTGAAAATTCTGGAGTTTCT<br>AGAAACTCCAGAATTTTCAAGCCAGAGGGATTCTCCACTTAATAAGCAGTTGATATAATT
c.7460_7489del		GAGAAGAACATGATATGTGGGTATTCCGACTTTCTGAAGTCAATGGCATGATGAAGGCAA<br>TTGCCTTCATCATGCCATTGACTTCAGAAAGTCGGAATACCCACATATCATGTTCTTCTC
c.7485_7512del		CCGACTTTGTTCCCTCTGGCTTGAAAATTCAAGGCAAGTGTTACTCAGCCCAATATTCTA<br>TAGAATATTGGGCTGAGTAACACTTGCCTTGAATTTTCAAGCCAGAGGGAACAAAGTCGG
c.7335_7344del		AAGATACACAGTAAAGGTTCAGCGAGAGCTGAATTAGCCCTGCGTGCACTGAAAGAGGAT<br>ATCCTCTTTCAGTGCACGCAGGGCTAATTCAGCTCTCGCTGAACCTTTACTGTGTATCTT
c.7343_7352del		CAGTAAAGGTTCAGCGAGAGCTGGAGTTGGCCTGCGTGCACTGAAAGAGGATCGTAAACG<br>CGTTTACGATCCTCTTTCAGTGCACGCAGGCCAACTCCAGCTCTCGCTGAACCTTTACTG
c.7351_7360del		GTTCAGCGAGAGCTGGAGTTGGATGAATTACACTGAAAGAGGATCGTAAACGCTTCTTAT<br>ATAAGAAGCGTTTACGATCCTCTTTCAGTGTAATTCATCCAACTCCAGCTCTCGCTGAAC
c.7359_7368del		AGAGCTGGAGTTGGATGAATTAGCCCTGCGGAGGATCGTAAACGCTTCTTATGTAAAGCA<br>TGCTTTACATAAGAAGCGTTTACGATCCTCCGCAGGGCTAATTCATCCAACTCCAGCTCT
c.-88_-59del	*BRIP1*	TATTTGCTCTCAGAAGTCGGTTTCCAGCGTTGCTTCGGTTTCCCTCCAGA<br>TCTGGAGGGAAACCGAAGCAACGCTGGAAACCGACTTCTGAGAGCAAATA
c.-63_-34del		TTTCCTTTTCTTCAGTGAATCGGAGCAGGCAAGTCGGAGGGAAGTCCACG<br>CGTGGACTTCCCTCCGACTTGCCTGCTCCGATTCACTGAAGAAAAGGAAA
c.-28_1del		TGACATTTTCATTTCTGTTGTAGTTGTCTTCAATGTGGTCTGAATATACA<br>TGTATATTCAGACCACATTGAAGACAACTACAACAGAAATGAAAATGTCA
c.-5_26del		AGGAATCTGAAATAAACAGGAAAGCATTGGTGGGGTGAAGATTTACTTTC<br>GAAAGTAAATCTTCACCCCACCAATGCTTTCCTGTTTATTTCAGATTCCT
c.3204_3233del	*PALB2*	TGGGATATTTATTTTTCTCCGAAATTAGGGTGCCAAAGAGAGTGAGTCGTTGCGAAGCCC<br>GGGCTTCGCAACGACTCACTCTCTTTGGCACCCTAATTTCGGAGAAAAATAAATATCCCA
c.3229_3258del		TAGGGGCTTCTCTTTATTGTCCTGAGTCATAGCCCTGTGTTTCAGCTCATTGTGATTAAC<br>GTTAATCACAATGAGCTGAAACACAGGGCTATGACTCAGGACAATAAAGAGAAGCCCCTA
c.3254_3297del		GTTAATCACAATGAGCTGAAACACAGGGCTATGACTCAGGACAATAAAGAGAAGCCCCTA<br>ACAGTACAGCATCACACCCACGCTGAGAGTACGACTCACTCTCTTTGGCACAGGGATGAC
c.3293_3322del		CTGTGTTTCAGCTCATTGTGATTAACCCTAACTGTCTTCCTCCAGGGCAGGCTGGCAGGC<br>GCCTGCCAGCCTGCCCTGGAGGAAGACAGTTAGGGTTAATCACAATGAGCTGAAACACAG
c.3318_3347del		CCCTAAGACGACTCTCAGCGTGGGTGTGATCAGGCAAGTGTGCATAACTGCTACTCTATG<br>CATAGAGTAGCAGTTATGCACACTTGCCTGATCACACCCACGCTGAGAGTCGTCTTAGGG
c.3229_3239del		GCTTCTCTTTATTGTCCTGAGTCATAGAGAGTGAGTCGTTGCGAAGCCCT

		CGGGCTTCGCAACGACTCACTCTCTATGACTCAGGACAATAAAFAGAAGC
c.3238_3248del		TATTGTCCTGAGTCATCCCTGTGCCGTCGTTGCGAAGCCCTGTGTTTCAG<br>CTGAAACACAGGGCTTCGCAACGACGGCACAGGGATGACTCAGGACAATA
c.3247_3258del		GAGTCATCCCTGTGCCAAAGAGAGTAGCCCTGTGTTTCAGCTCATTGTGA<br>TCACAATGAGCTGAAACACAGGGCTACTCTCTTTGGCACAGGGATGACTC
c.3293_3303del		TTTCAGCTCATTGTGATTAACCCTAAGCGTGGGTGTGATGCTGTACTGTC<br>GACAGTACAGCATCACACCCACGCTTAGGGTTAATCACAATGAGCTGAAA
c.3302_3312del		ATTGTGATTAACCCTAAGACGACTCGTGATGCTGTACTGTCTTCCTCCAG<br>CTGGAGGAAGACAGTACAGCATCACGAGTCGTCTTAGGGTTAATCACAAT
c.3311_3322del		AACCCTAAGACGACTCTCAGCGTGGACTGTCTTCCTCCAGGGCAGGCTGG<br>CCAGCCTGCCCTGGAGGAAGACAGTCCACGCTGAGAGTCGTCTTAGGGTT
**Variants**		**Primer (5’ →3’)**
c.7308-2A>C		TACCTTAATTATTCTATGCACGATACACAGTAAAGGTTCAG<br>CTGAACCTTTACTGTGTATCGTGCATAGAATAATTAAGGTA
c.7335G>W		AAAGGTTCAGCGAGAGCTWGAGTTGGATGAATTAGCC<br>GAGTTGGATGAATTAGCCWAGCTCTCGCTGAACCTTT
c.7336G>T		AAGGTTCAGCGAGAGCTGTAGTTGGATGAATTAGCCC<br>GGGCTAATTCATCCAACTACAGCTCTCGCTGAACCTT
c.7337A>K		AGGTTCAGCGAGAGCTGGKGTTGGATGAATTAGCCCT<br>AGGGCTAATTCATCCAACMCCAGCTCTCGCTGAACCT
c.7340T>R		TTCAGCGAGAGCTGGAGTRGGATGAATTAGCCCTGCG<br>CGCAGGGCTAATTCATCCYACTCCAGCTCTCGCTGAA
c.7341G>A	*ATM*	TCAGCGAGAGCTGGAGTTAGATGAATTAGCCCTGCGT<br>ACGCAGGGCTAATTCATCTAACTCCAGCTCTCGCTGA
c.7342G>H		CAGCGAGAGCTGGAGTTGHATGAATTAGCCCTGCGTG<br>CACGCAGGGCTAATTCATDCAACTCCAGCTCTCGCTG
c.7343A>B		AGCGAGAGCTGGAGTTGGBTGAATTAGCCCTGCGTGC<br>GCACGCAGGGCTAATTCAVCCAACTCCAGCTCTCGCT
c.7515+1G>T		AAGTCAATGGCATGATGAAGTCAAGTGTTACTCAGCCCAAT<br>ATTGGGCTGAGTAACACTTGACTTCATCATGCCATTGACTT
c.7515+2C>R		AGTCAATGGCATGATGAAGGRAAGTGTTACTCAGCCCAATA<br>TATTGGGCTGAGTAACACTTYCCTTCATCATGCCATTGACT
c.3202-1G>A		TTTATTTTTCTCCGAAATTAAGGGCTTCTCTTTATTGTCCT<br>AGGACAATAAAGAGAAGCCCTTAATTTCGGAGAAAAATAAA
c.3242A>G		CCTGTGCCAAAGGGAGTGAGTCGTTGCGAAGCCCTGTGTT<br>AACACAGGGCTTCGCAACGACTCACTCCCTTTGGCACAGG
c.3244A>G		GTCATCCCTGTGCCAAAGAGGGTGAGTCGTTGCGAAGCCC<br>GGGCTTCGCAACGACTCACCCTCTTTGGCACAGGGATGAC
c.3251C>T		CAAAGAGAGTGAGTTGTTGCGAAGCCCTGTGTTTCAGCTC<br>GAGCTGAAACACAGGGCTTCGCAACAACTCACTCTCTTTG
c.3251C>A		CTGAGTCATCCCTGTGCCAAAGAGAGTGAGTAGTTGCGAA<br>TTCGCAACTACTCACTCTCTTTGGCACAGGGATGACTCAG
c.3256C>G	*PALB2*	GTGAGTCGTTGGGAAGCCCTGTGTTTCAGCTCATTGTGAT<br>ATCACAATGAGCTGAAACACAGGGCTTCCCAACGACTCAC
c.3258A>T		AGTCGTTGCGTAGCCCTGTGTTTCAGCTCATTGTGATTAA<br>TTAATCACAATGAGCTGAAACACAGGGCTACGCAACGACT
c.3294_3298del		TGTTTCAGCTCATTGTGATTAACCCTAACTCTCAGCGTGG<br>CCACGCTGAGAGTTAGGGTTAATCACAATGAGCTGAAACA
c.3306C>G		CTAAGACGACTCTCAGGGTGGGTGTGATGCTGTACTGTCT<br>AGACAGTACAGCATCACACCCACCCTGAGAGTCGTCTTAG
c.3306C>T		AACCCTAAGACGACTCTCAGTGTGGGTGTGATGCTGTACT<br>AGTACAGCATCACACCCACACTGAGAGTCGTCTTAGGGTT

Supplementary Table S2. Splicing outcomes of *ATM*, *BRIP1* and *PALB2* microdeletions.

Microdeletions	Full-length Transcript	PTC-transcripts	In-frame transcripts	Uncharacterized
mgATM _49-52_WT[1]	73.3%±0.4%	[△(E50)△(E52)] [1.3%±0.6%]<br>△(E49p82) [1.6%]<br>△(E49q38) [1.7%±0.2%]	△(E52) [22.1% ±0.04%]	
c.7310_7339del	59.6%±1.0%	[△(E50)△(E52)] [15.3%±1.9%]	△(E52) [25.1%±2.9%]	
**c.7335_7364del**	16.4%±0.6%	△(E50) [33.9%±0.6%]<br>[△(E50)△(E52)] [49.7%±0.3%]		
**c.7335_7344del**	36.8%±0.5%	△(E50) [28.6%±0.6%]<br>[△(E50)△(E52)] [34.6%±0.8%]		
c.7343_7352del	83.2%±0.6%		△(E52) [16.8%±0.6%]	
c.7351_7360del	83.2%±0.4%		△(E52) [16.8%±0.4%]	
c.7359_7368del	85%±0.3%		△(E52) [15.0%±0.3%]	
c.7360_7389del	53%±1.0%	[△(E50)△(E52)] [25.7%±1.1%]<br>△(E50) [6.5%±0.1%]	△(E52) [14.8%±0.4%]	
c.7385_7414del	76.4%±1.1%		△(E52) [23.6%±1.1%]	
c.7410_7439del	64.1%±0.4%		△(E52) [35.9%±0.4%]	
c.7435_7464del	64.2%±2.3%		△(E52) [35.8%±2.3%]	
c.7460_7489del	58%±0.2%	△(E50) [5.2%]<br>[△(E50)△(E52)] [17.1%±0.1%]	△(E52) [19.7%±0.3%]	
c.7485_7512del	47.4%±1.7%	△(E50) [13.8%±1.9%]<br>[△(E50)△(E52)] [25.8%±1.5%]	△(E52) [13%±0.5%]	
mgPALB2_5-12_WT[1]	100%			
c.3204_3233del	100%			
**c.3229_3258del**	39.5%±1.2%	△(E12) [51.4%±0.7%]<br>△(E7p10) [1%±0.1%]<br>[△(E9)△(E12)] [5.6%±0.6%]		765-nt [2.5%±0.1%]
c.3229_3239del	92%±0.3%	Δ(E12) [8%±0.3%]		
c.3238_3248del	95%±0.3%	△(E7p10) [3.4%±0.4%]		728-nt [1.6%±0.1%]
c.3247_3258del	92.4%±0.7%	△(E12) [7.6%±0.7%]		
c.3254_3297del	92.5%±0.4%	△(E12) [7.5%±0.4%]		
**c.3293_3322del**	43.5%±0.9%	△(E12) [56.5%±0.9%]		
c.3293_3303del	86.4%±1.4%	△(E12) [6.1%±0.2%]<br>△(E7p10) [2.5%±0.4%]<br>[△(E9)△(E12)] [1%±0.1%]<br>[△(E10)△(E12)] [1.3%±0.1%]	△(E9) [1.5%±0.1%]	705-nt [1.2%±0.1%]
c.3302_3312del	93.8%±1.3%	△(E7p10) [2.8%±1.4%]	△(E9) [1.9%±0.1%]	705-nt [1.5%±0.2%]
c.3311_3322del	93.9%±0.8%	△(E12) [6.1%±0.8%]		
c.3318_3347del	100%			
mgBRIP1_1-2_WT[1]	68.8%±0.3%	△(E2p4) [31.2%±0.3%]		
c.-88_-59del (ex1)	NO IMPACT			
c.-63_-34del (ex1)				
c.-28_1del (ex2)				
c.-5_26del (ex2)				

[1]*ATM* exon 50 (208 bp: c.7308_7515); *PALB2* exon 12 (149 bp: c.3202_3350); *BRIP1* exon 1 (276 bp: 5'UTR: c.-306_-31). Deletions with the highest impacts are shown in bold.

Supplementary Table S3. DeepCLIP analysis of *ATM* and *PALB2* spliceogenic variants.

Splicing Factor	Model_id	Wild type-sequence	WT-Score	Variant	Variant sequence	Variant score	Δ score [1]	% FL-transcript
ATM								
SRSF1	RNCMPT00106_RNCMPT	agctggagttg	0.98	c.7336G>T	agctgtagttg	0.07	-0.91	
SRSF1	RNCMPT00108_RNCMPT	agctggagttg	0.96	c.7336G>T	agctgtagttg	0.08	-0.88	
SRSF1	RNCMPT00107_RNCMPT	agctggagttg	0.93	c.7336G>T	agctgtagttg	0.17	-0.76	
SRSF1	RNCMPT00110_RNCMPT	agctggagttg	0.86	c.7336G>T	agctgtagttg	0.25	-0.61	
HNRNPH2	RNCMPT00160_RNCMPT	agctggagttg	0.77	c.7336G>T	agctgtagttg	0.16	-0.61	
SRSF1	RNCMPT00109_RNCMPT	agctggagttg	0.70	c.7336G>T	agctgtagttg	0.14	-0.56	
SRSF9	RNCMPT00067_RNCMPT	agctggagttg	0.83	c.7336G>T	agctgtagttg	0.37	-0.46	
SRSF1	SRSF1_ENCODE-K562	agctggagttg	0.73	c.7336G>T	agctgtagttg	0.37	-0.36	55.4%
SRSF9	SRSF9_ENCODE-HepG2	agctggagttg	0.76	c.7336G>T	agctgtagttg	0.44	-0.33	
SRSF1	SRSF1_GP	agctggagttg	0.71	c.7336G>T	agctgtagttg	0.38	-0.33	
SRSF7	SRSF7_ENCODE-HepG2	agctggagttg	0.57	c.7336G>T	agctgtagttg	0.24	-0.32	
DAZAP1	RNCMPT00013_RNCMPT	agctggagttg	0.47	c.7336G>T	agctgtagttg	0.84	0.37	
PTBP1	RNCMPT00269_RNCMPT	agctggagttg	0.14	c.7336G>T	agctgtagttg	0.56	0.43	
PTBP1	RNCMPT00268_RNCMPT	agctggagttg	0.13	c.7336G>T	agctgtagttg	0.62	0.50	
TIA1	RNCMPT00077_RNCMPT	agctggagttg	0.38	c.7336G>T	agctgtagttg	0.93	0.54	
SRSF1	RNCMPT00106_RNCMPT	gctggagttgg	0.98	c.7337A>G	gctggggttgg	0.23	-0.75	
PCBP2	RNCMPT00044_RNCMPT	gctggagttgg	0.56	c.7337A>G	gctggggttgg	0.08	-0.48	
SRSF2	RNCMPT00072_RNCMPT	gctggagttgg	0.96	c.7337A>G	gctggggttgg	0.51	-0.45	50.7%
DAZAP1	RNCMPT00013_RNCMPT	gctggagttgg	0.55	c.7337A>G	gctggggttgg	0.14	-0.41	
SRSF9	RNCMPT00074_RNCMPT	gctggagttgg	0.88	c.7337A>G	gctggggttgg	0.47	-0.41	
TIA1	RNCMPT00077_RNCMPT	ggagttggatg	0.52	c.7340T>A	ggagtaggatg	0.19	-0.33	58.8%
DAZAP1	RNCMPT00013_RNCMPT	ggagttggatg	0.61	c.7340T>G	ggagtgggatg	0.11	-0.49	
TIA1	RNCMPT00077_RNCMPT	ggagttggatg	0.52	c.7340T>G	ggagtgggatg	0.02	-0.49	
SRSF9	RNCMPT00074_RNCMPT	ggagttggatg	0.82	c.7340T>G	ggagtgggatg	0.46	-0.36	61.8%
PCBP2	RNCMPT00044_RNCMPT	ggagttggatg	0.43	c.7340T>G	ggagtgggatg	0.08	-0.35	
SRSF7	RNCMPT00073_RNCMPT	ggagttggatg	0.31	c.7340T>G	ggagtgggatg	0.64	0.33	
SRSF1	SRSF1_GP	gagttggatga	0.77	c.7341G>A	gagttagatga	0.07	-0.69	40.4%

Splicing Factor	Model_id	Wild type-sequence	WT-Score	Variant	Variant sequence	Variant score	Δ score [1]	% FL-transcript
SRSF1	RNCMPT00108_RNCMPT	gagttggatga	0.99	c.7341G>A	gagttagatga	0.42	-0.57	
SRSF1	RNCMPT00106_RNCMPT	gagttggatga	1.00	c.7341G>A	gagttagatga	0.45	-0.54	
SRSF1	RNCMPT00110_RNCMPT	gagttggatga	0.74	c.7341G>A	gagttagatga	0.24	-0.50	
SRSF9	SRSF9_ENCODE-HepG2	gagttggatga	0.84	c.7341G>A	gagttagatga	0.34	-0.50	
SRSF9	RNCMPT00067_RNCMPT	gagttggatga	0.83	c.7341G>A	gagttagatga	0.39	-0.44	
SRSF1	SRSF1_ENCODE-HepG2	gagttggatga	0.80	c.7341G>A	gagttagatga	0.40	-0.40	
HNRNPM	HNRNPM_ENCODE-HepG2	gagttggatga	0.77	c.7341G>A	gagttagatga	0.38	-0.38	
SRSF1	RNCMPT00109_RNCMPT	gagttggatga	0.77	c.7341G>A	gagttagatga	0.42	-0.35	
HNRNPA2B1	RNCMPT00024_RNCMPT	gagttggatga	0.56	c.7341G>A	gagttagatga	0.96	0.40	
HNRNPL	RNCMPT00091_RNCMPT	gagttggatga	0.43	c.7341G>A	gagttagatga	0.91	0.48	
SRSF1	RNCMPT00110_RNCMPT	agttggatgaa	0.79	c.7342G>A	agttgaatgaa	0.19	-0.60	50.2%
SRSF1	RNCMPT00108_RNCMPT	agttggatgaa	0.99	c.7342G>A	agttgaatgaa	0.43	-0.56	
SRSF9	SRSF9_ENCODE-HepG2	agttggatgaa	0.83	c.7342G>A	agttgaatgaa	0.28	-0.55	
HNRNPH2	RNCMPT00160_RNCMPT	agttggatgaa	0.92	c.7342G>A	agttgaatgaa	0.46	-0.46	
SRSF1	SRSF1_ENCODE-HepG2	agttggatgaa	0.75	c.7342G>A	agttgaatgaa	0.34	-0.41	
SRSF1	SRSF1_GP	agttggatgaa	0.82	c.7342G>A	agttgaatgaa	0.43	-0.39	
SRSF1	SRSF1_ENCODE-K562	agttggatgaa	0.69	c.7342G>A	agttgaatgaa	0.33	-0.36	
HNRNPM	HNRNPM_ENCODE-HepG2	agttggatgaa	0.67	c.7342G>A	agttgaatgaa	0.36	-0.31	
SRSF1	RNCMPT00108_RNCMPT	agttggatgaa	0.99	c.7342G>T	agttgtatgaa	0.06	-0.93	51.3%
SRSF1	RNCMPT00106_RNCMPT	agttggatgaa	1.00	c.7342G>T	agttgtatgaa	0.15	-0.84	
SRSF1	RNCMPT00110_RNCMPT	agttggatgaa	0.79	c.7342G>T	agttgtatgaa	0.10	-0.68	
SRSF9	RNCMPT00067_RNCMPT	agttggatgaa	0.90	c.7342G>T	agttgtatgaa	0.24	-0.66	
SRSF1	RNCMPT00107_RNCMPT	agttggatgaa	0.98	c.7342G>T	agttgtatgaa	0.40	-0.58	
SRSF1	SRSF1_GP	agttggatgaa	0.82	c.7342G>T	agttgtatgaa	0.27	-0.55	
SRSF1	RNCMPT00109_RNCMPT	agttggatgaa	0.65	c.7342G>T	agttgtatgaa	0.10	-0.54	
HNRNPH2	RNCMPT00160_RNCMPT	agttggatgaa	0.92	c.7342G>T	agttgtatgaa	0.41	-0.51	
SRSF9	SRSF9_ENCODE-HepG2	agttggatgaa	0.83	c.7342G>T	agttgtatgaa	0.32	-0.50	
SRSF1	SRSF1_ENCODE-HepG2	agttggatgaa	0.75	c.7342G>T	agttgtatgaa	0.32	-0.43	
SRSF7	SRSF7_ENCODE-K562	agttggatgaa	0.72	c.7342G>T	agttgtatgaa	0.36	-0.36	
SRSF1	SRSF1_ENCODE-K562	agttggatgaa	0.69	c.7342G>T	agttgtatgaa	0.33	-0.36	
TRA2A	TRA2A_ENCODE-K562	agttggatgaa	0.71	c.7342G>T	agttgtatgaa	0.38	-0.33	
TRA2A	TRA2A_ENCODE-HepG2	agttggatgaa	0.78	c.7342G>T	agttgtatgaa	0.46	-0.32	

Splicing Factor	Model_id	Wild type-sequence	WT-Score	Variant	Variant sequence	Variant score	Δ score [1]	% FL-transcript
SRSF7	SRSF7_ENCODE-HepG2	agttggatgaa	0.59	c.7342G>T	agttgtatgaa	0.29	-0.30	
PTBP1	RNCMPT00269_RNCMPT	agttggatgaa	0.20	c.7342G>T	agttgtatgaa	0.67	0.47	
PTBP1	RNCMPT00268_RNCMPT	agttggatgaa	0.27	c.7342G>T	agttgtatgaa	0.77	0.50	
TIA1	TIA1_GP	gttggatgaat	0.89	c.7343A>G	gttgggtgaat	0.22	-0.67	49.4%
SRSF1	RNCMPT00106_RNCMPT	gttggatgaat	1.00	c.7343A>G	gttgggtgaat	0.42	-0.58	
SRSF1	RNCMPT00110_RNCMPT	gttggatgaat	0.75	c.7343A>G	gttgggtgaat	0.31	-0.44	
SRSF9	RNCMPT00067_RNCMPT	gttggatgaat	0.91	c.7343A>G	gttgggtgaat	0.48	-0.42	
SRSF1	SRSF1_ENCODE-K562	gttggatgaat	0.75	c.7343A>G	gttgggtgaat	0.35	-0.41	
SRSF9	SRSF9_ENCODE-HepG2	gttggatgaat	0.80	c.7343A>G	gttgggtgaat	0.45	-0.35	
SRSF1	RNCMPT00106_RNCMPT	gttggatgaat	1.00	c.7343A>T	gttggttgaat	0.22	-0.78	52.6%
SRSF1	RNCMPT00108_RNCMPT	gttggatgaat	0.99	c.7343A>T	gttggttgaat	0.35	-0.64	
SRSF1	RNCMPT00110_RNCMPT	gttggatgaat	0.75	c.7343A>T	gttggttgaat	0.17	-0.59	
SRSF1	RNCMPT00107_RNCMPT	gttggatgaat	0.98	c.7343A>T	gttggttgaat	0.48	-0.50	
SRSF1	RNCMPT00109_RNCMPT	gttggatgaat	0.62	c.7343A>T	gttggttgaat	0.13	-0.49	
TIA1	TIA1_GP	gttggatgaat	0.89	c.7343A>T	gttggttgaat	0.40	-0.49	
SRSF1	SRSF1_ENCODE-K562	gttggatgaat	0.75	c.7343A>T	gttggttgaat	0.27	-0.48	
SRSF9	RNCMPT00067_RNCMPT	gttggatgaat	0.91	c.7343A>T	gttggttgaat	0.44	-0.46	
SRSF9	SRSF9_ENCODE-HepG2	gttggatgaat	0.80	c.7343A>T	gttggttgaat	0.39	-0.41	
SRSF1	SRSF1_ENCODE-HepG2	gttggatgaat	0.71	c.7343A>T	gttggttgaat	0.36	-0.35	
TRA2A	TRA2A_ENCODE-HepG2	gttggatgaat	0.75	c.7343A>T	gttggttgaat	0.42	-0.33	
SRSF1	SRSF1_GP	gttggatgaat	0.97	c.7343A>T	gttggttgaat	0.66	-0.32	
PALB2								
HNRNPL	RNCMPT00027_RNCMPT	caaagagagtg	0.69	c.3242A>G	caaagggagtg	0.21	-0.49	89.7%
DAZAP1	RNCMPT00013_RNCMPT	caaagagagtg	0.81	c.3242A>G	caaagggagtg	0.33	-0.47	
PCBP2	RNCMPT00044_RNCMPT	caaagagagtg	0.82	c.3242A>G	caaagggagtg	0.40	-0.42	
SRSF1	RNCMPT00110_RNCMPT	caaagagagtg	0.49	c.3242A>G	caaagggagtg	0.86	0.36	
SRSF1	RNCMPT00108_RNCMPT	caaagagagtg	0.56	c.3242A>G	caaagggagtg	0.99	0.43	
HNRNPA2B1	RNCMPT00024_RNCMPT	caaagagagtg	0.53	c.3242A>G	caaagggagtg	0.99	0.46	
SRSF7	RNCMPT00073_RNCMPT	cctaagacgactctc	0.83	c.3294_3298del	accctaactctcagc	0.04	-0.79	83.1%
SRSF1	RNCMPT00106_RNCMPT	cctaagacgactctc	0.75	c.3294_3298del	accctaactctcagc	0.01	-0.74	
TRA2B	TRA2B_GSE59335	cctaagacgactctc	0.62	c.3294_3298del	accctaactctcagc	0.12	-0.50	
SRSF5	SRSF5_GSE113813	cctaagacgactctc	0.73	c.3294_3298del	accctaactctcagc	0.28	-0.45	

Splicing Factor	Model_id	Wild type-sequence	WT-Score	Variant	Variant sequence	Variant score	Δ score [1]	% FL-transcript
SRSF1	RNCMPT00109_RNCMPT	cctaagacgactctc	0.48	c.3294_3298del	accctaactctcagc	0.03	-0.45	
SRSF1	RNCMPT00107_RNCMPT	cctaagacgactctc	0.45	c.3294_3298del	accctaactctcagc	0.01	-0.44	
SRSF7	SRSF7_ENCODE-K562	cctaagacgactctc	0.69	c.3294_3298del	accctaactctcagc	0.25	-0.43	
SRSF10	RNCMPT00019_RNCMPT	cctaagacgactctc	0.50	c.3294_3298del	accctaactctcagc	0.10	-0.40	
SRSF1	RNCMPT00110_RNCMPT	cctaagacgactctc	0.54	c.3294_3298del	accctaactctcagc	0.14	-0.39	
SRSF6	SRSF6_GSE113813	cctaagacgactctc	0.86	c.3294_3298del	accctaactctcagc	0.50	-0.36	
SRSF10	RNCMPT00089_RNCMPT	cctaagacgactctc	0.40	c.3294_3298del	accctaactctcagc	0.04	-0.36	
SRSF9	SRSF9_ENCODE-HepG2	cctaagacgactctc	0.55	c.3294_3298del	accctaactctcagc	0.21	-0.34	
SRSF9	RNCMPT00067_RNCMPT	cctaagacgactctc	0.46	c.3294_3298del	accctaactctcagc	0.12	-0.34	
SRSF1	SRSF1_ENCODE-K562	cctaagacgactctc	0.64	c.3294_3298del	accctaactctcagc	0.31	-0.33	
TRA2A	TRA2A_ENCODE-HepG2	cctaagacgactctc	0.51	c.3294_3298del	accctaactctcagc	0.18	-0.33	
SRSF1	RNCMPT00108_RNCMPT	cctaagacgactctc	0.33	c.3294_3298del	accctaactctcagc	0.00	-0.32	
SRSF1	SRSF1_GP	cctaagacgactctc	0.31	c.3294_3298del	accctaactctcagc	0.62	0.31	
TIA1	RNCMPT00165_RNCMPT	cctaagacgactctc	0.21	c.3294_3298del	accctaactctcagc	0.61	0.40	
SRSF2	RNCMPT00072_RNCMPT	ctcagcgtggg	0.78	c.3306C>G	ctcagggtggg	0.38	-0.40	
SRSF7	SRSF7_ENCODE-K562	ctcagcgtggg	0.70	c.3306C>G	ctcagggtggg	0.31	-0.40	
HNRNP L	RNCMPT00091_RNCMPT	ctcagcgtggg	0.93	c.3306C>G	ctcagggtggg	0.55	-0.38	
TIA1	TIA1_GP	ctcagcgtggg	0.91	c.3306C>G	ctcagggtggg	0.58	-0.32	
SRSF1	SRSF1_ENCODE-K562	ctcagcgtggg	0.60	c.3306C>G	ctcagggtggg	0.28	-0.32	
SRSF1	RNCMPT00108_RNCMPT	ctcagcgtggg	0.66	c.3306C>G	ctcagggtggg	0.99	0.33	49.3%
SRSF1	RNCMPT00110_RNCMPT	ctcagcgtggg	0.39	c.3306C>G	ctcagggtggg	0.73	0.35	
HNRNPA1	RNCMPT00022_RNCMPT	ctcagcgtggg	0.59	c.3306C>G	ctcagggtggg	0.95	0.37	
SRSF1	RNCMPT00107_RNCMPT	ctcagcgtggg	0.46	c.3306C>G	ctcagggtggg	0.88	0.42	
HNRNPA1L2	RNCMPT00023_RNCMPT	ctcagcgtggg	0.37	c.3306C>G	ctcagggtggg	0.84	0.47	
SRSF1	RNCMPT00106_RNCMPT	ctcagcgtggg	0.11	c.3306C>G	ctcagggtggg	0.62	0.51	

[1] Gains and losses in the binding capacity of splicing factors are shown in green and red, respectively.

Supplementary Table S4. AlphaMissense and REVEL scores of *ATM* and *PALB2* spliceogenic missense variants.

Variants	AlphaMissense[1]	REVEL[2]
ATM		
c.7337A>G p.(Glu2446Gly)	0.142 (likely_benign)	0.617
c.7340T>G p.(Leu2447Trp)	0.397 (ambiguous)	**0.861 (PP3)**
c.7342G>A p.(Asp2448Asn)	**0.898 (likely_pathogenic)**	0.662
c.7342G>T p.(Asp2448Tyr)	**0.923 (likely_pathogenic)**	**0.893 (PP3)**
c.7343A>G p.(Asp2448Gly)	**0.902 (likely_pathogenic)**	**0.863 (PP3)**
c.7343A>T p.(Asp2448Val)	**0.960 (likely_pathogenic)**	**0.921 (PP3)**
PALB2		
c.3306C>G p.(Ser1102Arg)	0.623 (likely_pathogenic)	0.102 (BP4)

[1]AlphaMissense: likely benign, 0-0.34; ambiguous, 0.34-0.564; likely pathogenic, 0.564-0.78; likely pathogenic, 0.78-1.0.
[2]HBOPC_ATMv1 specifications support PP3 (pathogenic supporting) code for REVEL meta-predictor score >.773 and BP4 (benign supporting evidence) for REVEL score <.249.
